# Supplementary material for: An effective spatial relational reasoning networks for visual question answering
Source: PLoS One. 2022 Nov 28;17(11):e0277693. doi: 10.1371/journal.pone.0277693 (PMC9704574; doi:10.1371/journal.pone.0277693)
Supplement: Attachment — Submitted filename: Response to Reviewers.docx [file pone.0277693.s001.docx]

**Manuscript ID:**  PONE-D-21-39541

**Paper Title:** “An effective spatial relational reasoning networks for visual question answering”

Dear Editor,

Thanks very much for taking your time to review this manuscript. We really appreciate all your generous comments and suggestion, which are very helpful to make this revision much clearer and more compelling. We have followed the comments and suggestions and revised the manuscript.

Thanks again for your patience and guidance!

Best regards,

Xiang Shen, Dezhi Han, Chongqing Chen, Gaofeng Luo, Zhongdai Wu

**Journal Requirements Response:**

1. Thank you very much for pointing out the format of this article. In accordance with the format requirements of the Plos One journal, the format of the article has been modified. According to journal requirements, we also invite peer English-speaking professionals to polish and revise the manuscript. Grammatical errors in the manuscript have been corrected and are highlighted in **red** throughout the manuscript.

2. Modification to the third suggestion: the content of the **Acknowledgments** section has been deleted. Also add the role of author participation at the end of the manuscript. Research grant funding is also updated and stated in the **cover letter**.

3. As for the fifth suggestion, the copyright issue in parts of Figure 1, 2 and 8 is because the images in the data set selected for the model are copyrighted. These data sets are public and free to use, and the license certificate is placed in the "others" file.

**Response to Editor**

**Additional Editor Comments (if provided):
The paper is based on the traditional approach to VQA with 71% accuracy. The recent approaches (pre-trained Transformer) have achieved performance of ~81% accuracy. The authors need to discuss those approaches and how (in terms of computational complexity, interoperability, low resource scenarios, etc. ) and when the proposed method will be useful.**

**Response editor：**

First of all, thank you very much for your comment. Pre-training is widely used in vision and language tasks, achieving surprising results in various tasks. Undoubtedly, pre-training can effectively facilitate alignment between different modalities, and reasonable fine-tuning in downstream tasks can achieve great results. It has reached 81% in the VQA task.

Although the pre-trained model achieves good results in a specific VQA task, many researchers also focus on developing end-to-end models. For public VQA benchmark datasets, there are VQA 1.0, VQA 2.0, GQA, VQA-E, CLEVR, etc. To verify the effectiveness of the proposed models, researchers usually use these benchmarks dataset for training. We employ end-to-end development because we can flexibly design and change the network structure without considering the effect of the model due to dataset bias or other factors. In addition, considering the hardware resources, if a pre-trained model is used to obtain a pre-trained model for a specific VQA task, the amount of datasets and better hardware resources are required for training. Currently, our hardware resources are insufficient to support pre-training, and pre-training also takes a long time. The final model is fine-tuned on the VQA task to achieve good results. If we directly utilize the existing pre-trained model and fine-tune it in a specific VQA task, it may also be due to the bias of the dataset and the differences between tasks. Without an excellent fine-tuning strategy, the final result may also be unsatisfactory. Since we changed the model structure of the network, we cannot rigorously prove the effectiveness of our model without using a specific dataset for training.

Of course, we are also considering using pre-trained models in future work. To ensure that our end-to-end model has a perfect effect, continuing to employ pre-training for fine-tuning will promote the development of the VQA task and get better results. In recent research work, there are also pre-trained models using similar network structures in this paper, such as ViLT-BERT, VisualBERT, and ViLBERT, among which the accuracies of ViBERT, VisualBERT, and ViLBERT are 70.34%, 70.80%, 70.55%, respectively. The SRRN model is better than these models.

Based on your comments, to allow readers to understand our research work better, we have added pre-trained models and explained them in the **related work** section and model comparison. Related pre-trained models have been discussed in the related work section (Section 2.1: lines134-159) and the model comparison section (Section 4.4: lines 611-615), with modifications marked in **blue**.

**Response to Reviewer #1**

Dear Reviewer#1:

Thank you for reviewing our paper entitled “An effective spatial relational reasoning networks for visual question answering. (ID:PONE-D-21-39541)” Also, we would like to thank you for your good comments and suggestions on it. We have studied each of your comments carefully and made corresponding revisions at your suggestion, which are highlighted in **blue** in this paper. All of your question were answered one-by-one.

**Reviewer #1: This work introduces the spatial and semantic reasoning network to learn the spatial position relationship and object attribute relationship of visual objects in VQA tasks. The authors show that their approach outperforms the attention and fusion-based existing methods on VQA 2.0 and GQA. However, there is no discussion on ongoing pre-trained Transformer based approaches. The pre-trained networks have pushed the boundaries of VQA performance. A detailed discussion on pre-trained networks and why one should use the proposed method (with less than ~10% overall accuracy compared to pre-trained approaches) is required for the reader to understand the usefulness of the approach.**

**Response：**

First of all thank you very much for your comment, as you said, “The pre-trained networks have pushed the boundaries of VQA performance.” In the past few years, the emergence of pre-training models has brought uni-modal fields such as computer vision and natural language processing to a new era. Substantial works have shown they are beneficial for downstream uni-modal tasks and avoid training a new model from scratch. Pre-training is an important technology in two aspects of computer vision and natural language, whose models can fine-tune different downstream tasks. Some representative methods, including ViLBERT, VLBERT, LXMERT, UNITER, OSCAR, VisualBERT, etc., use the bidirectional encoder representations from Transformers (BERT) structure and have achieved effective results in VQA tasks.

Why do we consider using an end-to-end network structure?

First, to demonstrate the SRRN model's effectiveness, we conveniently use the traditional end-to-end model to compare with the baseline model MCAN. The end-to-end model can be trained and tested on specific datasets (VQA 2.0 and GQA).

Second, for a specific VQA task. We train with a benchmark dataset for the VQA task, which can illustrate the rationality and effectiveness of the model more effectively. Most of the vision and language tasks that use pre-training today include multiple tasks, such as masked language modeling, masked object prediction (feature regression and label classification), cross-modality matching, and image question answering. Fine-tuning for downstream tasks requires meeting the demands of multiple tasks, and enabling parameter sharing is also a challenge.

Finally, although pre-trained models can achieve outstanding results on some tasks, there are certain disadvantages to using pre-trained models. For example, the pre-training model is large, the parameters are many, the flexibility of the model structure is poor, it is difficult to change the network structure, the calculation amount is large, and the application scenarios are limited.

Based on your comments, to allow readers to understand our research work better, we have added pre-trained models and explained them in the related work section and model comparison. Related pre-trained models have been discussed in the related work section (Section 2.1: lines 134-159) and the model comparison section (Section 4.4: lines 611-615), with modifications marked in **blue**.

**Strengths:
The results show their approach improves the performance on the VQA 2.0 and GQA datasets compared to the non-pretrained network.
A detailed experiment and analysis are provided.

Weakness:
The paper proposed exciting ideas, but they can be presented in a much better way.
Related work on pre-trained network-based approaches and comparisons are missing.**

**Response：**First of all, thank you very much for your affirmation of our research work and for pointing out the problems in our manuscript. In response to the issues in the manuscript, we carefully revise and improve it according to your comments, and it is your comments that make our research work and manuscripts be presented to readers in a better way.

**Questions:

Q1. With n words in the question, Eq. (3) must obey the index. It can not be n+1. Please re-write the equation.**

**Response Q1:**Thank you very much for pointing out the problems in our manuscript. Based on your comments, we re-checked the manuscript. To give readers a better understanding of the formulas in the manuscript, we use *S* to represent the maximum number of words so as not to conflict with *n* in Equation 3. Modifications in the manuscript are marked in **blue** as lines 264 to 269. Modified Equations and symbols are marked in **yellow**.

**Q2. It is written (line 243-245) “Therefore, we use the question-adaptive attention mechanism to extract the semantic information of the question when designing the visual object spatial relationship graph reasoning network. “ However, the question-information is never incorporated to generate the reasoning feature v* of spatial object relation. Please clarify.**

**Response Q2:** First of all, thank you very much for your comments. Section 3.2 mainly describes the visual object spatial relation inference module. The original meaning of what we want to express is: that the visual object spatial relationship combined with the visual semantic reasoning module can obtain image features with spatial relationships and object semantic attributes.The question information is not used in the spatial object reasoning module. Only in the semantic relation reasoning module is semantic information utilized to guide the attention to the vital information of the image. The sparse attention mechanism in the visual object semantic reasoning module can adaptively obtain critical visual semantic features according to the question information. In order not to cause misunderstanding and ambiguity to the readers, according to your suggestion, we remove the description of the information about the semantic reasoning of visual objects in this section3.2 (lines292-294).

**Q3. Function M in Eq. 10 is independent of the δ. Please re-write it. Authors should be consistent with the notation. The variable Q denotes multiple things (question and key) in the paper.**

**Response Q3:** First of all, thank you very much for your comment, based on your comments. To allow readers to understand the meaning of the equation(10) better, we have re-worked the equation in the manuscript. Specifically: Rewrite the formula in Eq. 10, delete δ. Redefine the vector-matrix A. Modifications in the manuscript are marked in **yellow** as lines 369 to 372.

According to your comments, *Q* appears several times in the manuscript because *Q*, *K*, and *V* are used in both the encoder and decoder. To allow readers to understand *Q's* meaning in each chapter clearly, we use different subscripts to indicate different meanings. In the revised manuscript, *Q* used in the encoder is changed to *Q_E_*, and *V* and *K* are also represented in the same way. *Q*, *K*, and *V* also appear in Section 3.3.2. To illustrate the compact self-attention principle, we still use the original dot-product self-attention mechanism formulation method because the compact self-attention(CSA) mechanism also uses the encoder and decoder. Modifications in the manuscript are marked in **yellow** as Line352-350 and lines 380-387. In addition, the question feature vector is represented by *Q_q_*.

**Q4. Eq. 11 and 12 are the same, and the description around them are hard to follow. If the matrix W has c row (ref. Line 309), then how come the vector t has t (again, please use another notation) row as written in “We connect the thresholds of each row to form a vector t = [t1, t2, . . . , tt ]”?**

**Response Q4:** First of all, thank you very much for your comments. I am very sorry for the inconvenience caused to your review due to the repeated formulas caused by our carelessness in typesetting. Based on your comments. We have re-changed the repeated equation (12) and made the definition and interpretation of the equation.To make the formulas in the manuscript concise and easy to understand, we also rewrite the formulas that share the same symbols and re-denote the vector-matrix originally represented by *t* in the manuscript with ***A***. And also modify **fig4** in lines 369-370.

**Q5. The function SAtt(;) is not defined. Please define and refer to the appropriate equation/section. Can provide the appropriate reference to your statement: “Since Q and K vary linearly from the n yousame input, the weight matrices Wk and WQ are entangled in the gradient backpropagation, a basic redundancy in the conventional self-attention mechanism”.**

**Response Q5:** Thank you very much for your comment. Based on your comment, we have redefined the formula SAtt(;) in line389. Besides, our proposed compact self-attention mechanism is also inspired by the literature **[21]**, and the cited reference in line 398 of the manuscript explains the compact self-attention mechanism(CSA). In the VQA task, we have found this simple but very effective method through many experiments. So we use this method in this paper, which can also inspire readers to use the compact self-attention mechanism to improve the model accuracy in similar VQA tasks.

**Q6. What do the authors mean by this statement: line 628
“This paper proves that SRRN is indeed a virtual visual object spatial relationship network.”**

**Response Q6:** Thank you very much for pointing out that sentences in our manuscript are complicated for readers to understand, based on your prompt. We rechecked and revised, and rewritten the **Conclusion** section. Sections re-written in the manuscript's **Conclusions** are marked in **blue**.

**Q7. The contributions statement needs to be re-written. It is hard for the reader to understand.What does this sentence refer to: “The ablation experiment analyzes the hypernatremia effect of the SSRN model.”**

**Response Q7:** Thank you very much for your comments, based on your comments. For readers to better read and understand our research work, we re-written the contributions of this paper. Modifications in the manuscript are marked in **blue** as lines 104 to 118.

**Q8. What is vg in Section 4.3?**

**Response Q8:**Visual genome (*vg*) is a dataset, a knowledge base, an ongoing effort to connect structured image concepts to language. We have explained the VG dataset in lines 528 and 529 of the manuscript.To effectively verify the experiment, three modes can be used during the experiment：--SPLIT={'train', 'train+val', 'train+val+vg'} can combine the training datasets. The default training split is 'train+val+vg'. Setting --SPLIT='train' will trigger the evaluation script to run the validation score after every epoch automatically. The download address of the *vg* dataset : <https://pan.baidu.com/s/1QCOtSxJGQA01DnhUg7FFtQ#list/path=%2F>

**Q9. In Table 1, the performance of SRRN-c is better than SRNN-s. Does the proposed sparse attention mechanism not better than the traditional one?**

**Response Q9:** First of all, thank you very much for your comments. In **Table 1**, "SRRN-c" is the experimental effect when we only utilize the compact self-attention mechanism without employing the sparse self-attention encoder and the spatial position relationship reasoning module. From the experimental results, It can be seen that although the compact self-attention mechanism has a simple idea, it has a good effect on improving the model's performance. Our baseline model is MCAN, and it can be seen from Table 1 that the impact of using a sparse attention mechanism (SRRN-s) in the encoder is better than that of the MCAN model, indicating that our proposed sparse attention mechanism is effective.

Additionally, the main goal of our research is to explore the use of suitable parameters in sparse self-attention combined with visual object spatial relational reasoning modules. As shown in Table 1, when only the compact self-attention mechanism is used, the change in the "Number" indicator is not apparent, although the overall effect is good. However, our proposed visual object spatial relationship reasoning aims to study the spatial position relationship between objects and objects, so improving the "Number" indicator in the experimental results is needed to prove the method's effectiveness.Therefore, we combine the sparse attention mechanism and the visual object spatial relation inference module for research, and the experimental results also demonstrate the effectiveness of our two methods. Finally, the model can be further optimized if the compact self-attention mechanism is added. The Number indicator of "SRRN-r1-s-c" in Table 1 has reached 55.02%, which has surpassed some models that focus on counting (Number), such as ReGAT [20 ] and BAN-Counter [15] et al.

**Q10. Each model variant can be better presented in the itemized format.**

**Response Q10：**Thank you very much for your comments, based on your comments. We re-arrange and breakdown the data results for **Table 1**.

**Q11. “We input the word embedding sequence of size n × 300 to get the question, where n × 300 is the number of words contained in the question.” I think n × 300 should be changed to n.**

**Response Q11：**First of all, thank you very much for checking the manuscript carefully and carefully for us. I am sorry that there are errors in the manuscript due to our hand mistakes. According to your suggestion, for readers to understand the steps of our research very clearly. We have reworked the content, marked in yellow (line 264-269 marked **blue**).

**Q11. “Inattention will lead to the failure of relevant information extraction.” Duplicate sentence line 348-351.**

**Response Q11:** Thank you very much for pointing out the problems in our manuscript, based on your hints. We checked and rechecked the manuscript, and now repeated sentences have been removed, and ambiguous sentences have been rewritten. The specific revisions have been marked in **blue** (line342-343) the manuscript. Repeated sentences on lines 348-351 have been re-corrected.

**Response to Reviewer #2**

**Reviewer #2: Good
1. The research background is clearly articulated.
2. Distinct progress is shown at the paper.**

Dear Reviewer#2:

First of all, thank you very much for your affirmation and encouragement of our research work, and thank you for reviewing our paper entitled “An effective spatial relational reasoning networks for visual question answering. (ID:PONE-D-21-39541)”. We are very sorry for the inconvenience caused to your review due to some defects in our article. We re-checked the manuscript and ensured that all data and formulas were utterly correct. Again, thank you very much for your affirmation of our work.

**Q1. Results and Dataset are not available in the specified paths. (https://eval.ai/web/challenges/challengepage/830/submission)**

**Response Q1:** Thank you very much for your comments, based on your comments. To ensure the accuracy of our experimental data, we upload our experimental code and some test models to the Baidu network disk. Among them, the VQA 2.0 dataset evaluation website: https://eval.ai/auth/login **Username: shenxiang Password: 123456** **VQA 2.0** dataset website:：<https://pan.baidu.com/s/1C7jIWgM3hFPv-YXJexItgw#list/path=%2F>

The data underlying the results presented in the study are available from (include the name of the third party https://visualqa.org/vqa_v2_teaser.html).

**Q2. The architecture & training of Graph Neural Network can be elaborated.**

**Response Q2:** Thank you very much for your comment. In this paper, we use the principles of graph neural networks in our spatial object-relational reasoning network. Specifically, we use the different visual object targets in the image as different nodes as the graph neural network as input and the relationship between different objects as the edge as the graph neural network. It is explained in Section 3.2 of the manuscript how to calculate the relation weights between different objects and finally get visual features with spatial relation positions.

**Q3. Lines 348~351 are repeated.**

**Response Q3:** Thank you very much for your careful examination of our manuscript, according to your request. We have removed duplicate sentences.
