# Supplementary material for: An effective spatial relational reasoning networks for visual question answering
Source: PLoS One. 2022 Nov 28;17(11):e0277693. doi: 10.1371/journal.pone.0277693 (PMC9704574; doi:10.1371/journal.pone.0277693)
Supplement: Attachment — Submitted filename: Response to Reviewers.docx [file pone.0277693.s002.docx]

**Manuscript ID:** PONE-D-21-39541R1

**Paper Title:** An effective spatial relational reasoning networks for visual question answering

Dear Editor,

Thank you for your letter and for the reviewers’ comments concerning our manuscript entitled “An effective spatial relational reasoning networks for visual question answering” (PONE-D-21-39541R1). These comments are all valuable and very helpful for revising and improving our paper, as well as the important guiding significance to our research. We have carefully studied the comments point-by-point and revised the paper accordingly.

Thanks again for your patience and guidance!

Best regards,

Xiang Shen, Dezhi Han, Chongqing Chen, Gaofeng Luo, Zhongdai Wu

**Journal Requirements Response:**

**Please review your reference list to ensure that it is complete and correct. If you have cited papers that have been retracted, please include the rationale for doing so in the manuscript text, or remove these references and replace them with relevant current references. Any changes to the reference list should be mentioned in the rebuttal letter that accompanies your revised manuscript. If you need to cite a retracted article, indicate the article’s retracted status in the References list and also include a citation and full reference for the retraction notice.**

**Response:** First of all, thank you very much for your comments. According to the journal requirements, we rechecked the references cited in this article. The references we cite are not retracted papers. In addition, according to the requirements of reviewer #1, we re-added the pre-training model related to this paper and compared the parameter quantity and accuracy of the SRRN model proposed in this paper. For the convenience of reviewing, the newly added references are marked in yellow.

Reviewer's Responses to Questions

**Comments to the Author**
**1. If the authors have adequately addressed your comments raised in a previous round of review and you feel that this manuscript is now acceptable for publication, you may indicate that here to bypass the “Comments to the Author” section, enter your conflict of interest statement in the “Confidential to Editor” section, and submit your "Accept" recommendation.**

Reviewer #1: (No Response)

Reviewer #2: All comments have been addressed

**Response:** Thanks again to the editors and reviewers for their comments and suggestions on our paper. We carefully thought about and responded to all the questions raised, and marked them with different colors in the manuscript for easy review.

**2. Is the manuscript technically sound, and do the data support the conclusions?

The manuscript must describe a technically sound piece of scientific research with data that supports the conclusions. Experiments must have been conducted rigorously, with appropriate controls, replication, and sample sizes. The conclusions must be drawn appropriately based on the data presented.**

Reviewer #1: Yes

Reviewer #2: Yes

**Response:** I am very grateful for your affirmation of our work.

**3. Has the statistical analysis been performed appropriately and rigorously?**

Reviewer #1: Yes

Reviewer #2: N/A

**Response：**Thank you very much for reviewing and commenting on our paper again, in order to allow readers to better understand the method we propose. Based on editor and reviewer comments, we further refined the article and marked it in the manuscript.

**4. Have the authors made all data underlying the findings in their manuscript fully available?
The [PLOS Data policy](http://www.plosone.org/static/policies.action#sharing) requires authors to make all data underlying the findings described in their manuscript fully available without restriction, with rare exception (please refer to the Data Availability Statement in the manuscript PDF file). The data should be provided as part of the manuscript or its supporting information, or deposited to a public repository. For example, in addition to summary statistics, the data points behind means, medians and variance measures should be available. If there are restrictions on publicly sharing data—e.g. participant privacy or use of data from a third party—those must be specified**.

Reviewer #1: Yes

Reviewer #2: No

**Response：**Thank you very much for your review and comments on our article again. In order to allow readers to better understand and reproduce our proposed method, and conduct in-depth research, The code will be available at https://github.com/shenxiang-vqa/SRRN.

**5. Is the manuscript presented in an intelligible fashion and written in standard English?
PLOS ONE does not copyedit accepted manuscripts, so the language in submitted articles must be clear, correct, and unambiguous. Any typographical or grammatical errors should be corrected at revision, so please note any specific errors here.**

Reviewer #1: Yes

Reviewer #2: Yes

**Response：**I am very grateful for your affirmation of our work.

**6. Review Comments to the Author
Please use the space provided to explain your answers to the questions above. You may also include additional comments for the author, including concerns about dual publication, research ethics, or publication ethics. (Please upload your review as an attachment if it exceeds 20,000 characters)**

**Reviewer #1: I read the author's response to the pre-trained models' question. The computational resource issue is a valid concern for academia. But again, many of the models do not take many resources (GPUs) and time only to fine-tune them on a particular dataset. Does it mean the author proposed model is less complex and has fewer parameters than the existing pre-trained models?
In this case, it will be good to compare parameters and time taken to train (fine-tune) the model (if computational resources allow) between the proposed model and pre-trained models such as ViLBERT, VisualBERT, etc.**

**Response:** First of all, thank you very much for reviewing our paper again and giving us suggestions. Based on your suggestions and comments, we can better improve the article. As you said, most of the existing multi-modal pre-training models only need to be fine-tuned on specific task datasets to get good results, and pre-training models are gradually becoming the mainstream method for multi-modal tasks.

Based on your suggestion, we investigate and study the task of pretraining models for visual question answering. Through investigation and research, it is found that pre-trained models have their advantages. However, some pre-trained models have more parameters than our proposed (SRRN) model, and they are not as good as end-to-end models on visual question answering tasks. As shown in Table 1, we compare the existing classical visual question answering pre-training model with the SRRN model, and the SRRN model has advantages over the pre-training model in terms of both the amount of parameters and the accuracy. Through experimental comparison, it is found that we use the end-to-end model not only in terms of the number of parameters and far lower than the pre-trained model, but only 1 TitanX GPUs are required to train the model. It shows that the parameters and complexity of our proposed model are better than some existing pre-trained models. (Tabular data refer to **Table 1** in the “*Multi-stage Pre-training over Simplified Multimodal Pre-training Models*”.)

| model | parameter | Test-dev | Test-std |
| --- | --- | --- | --- |
| Unified VLP | - | 70.50 | 70.70 |
| VilBERT | 218.9M | 70.55 | 70.92 |
| VisualBERT | 85.05M | 70.80 | 71.00 |
| VL-BERT | 134.8M | 71.16 | - |
| DFAF-BERT | 173.2M | 70.59 | 70.81 |
| MLI-BERT | 120.0M | 71.19 | 71.27 |
| SRRN-r2-s | 58.98M | 70.73 | - |
| SRRN-r1-s | 58.19M | 70.92 | 71.18 |

According to your comment, we added the model parameters and model accuracy in the ablation experiment part of the paper (**Section 4.3.3**) to compare with some existing VQA pre-training models. Although the proposed model adds the spatial object relation inference module, it does not increase the parameter quantity and complexity of the model, and good results can be obtained by training under the same experimental conditions. The parameters trained by the two models are shown in the figure below, where the left side represents the parameter quantity for training the "SRRN-r1-s" model, and the right side represents the parameter quantity for training the "SRRN-r2" model. **Section 4.3.3** that we have added to the manuscript are marked in light blue.

**The dataset bias issue with the pre-trained model also needs to be discussed in the main paper.**

**Response：**First of all，thank you very much for your question, based on your suggestion. We discussed the issue of dataset bias for pretrained models in the paper. The pre-trained datasets come from various corpora, and the features learned from different corpora have generalization and generality, and can be better used for different multimodal tasks after fine-tuning. We use an end-to-end model for visual question answering tasks through specific datasets (such as VQA 2.0 and GQA), making it easier to capture and extract text and image features, increasing the robustness of the model. Re-added in **Section 4.1**, in lines **443 to 450** of the manuscript.

**Line 158 "....many researchers are still based on the end-to-end training method..." spelling mistakes and appropriate citations are missing.**

**Response**：First of all, I am very sorry for the spelling mistakes in the manuscript due to our carelessness, and thank you very much for pointing out our problems. Based on your suggestion, we carefully check and correct the errors in the manuscript. Re-edited content in lines **129 to 132** of the manuscript.

**The remaining concerns have been addressed. However, proofreading is required, and the uses of notation and equations need to be checked carefully.**

Reviewer #2: (No Response)

**Response**：Thank you again for your careful review and comments on our paper, based on your suggestions. We again carefully check the manuscript to ensure the correctness of the manuscript.
